# “Who Champions or Mentors Others”? The Role of Personal Resources in the Perceived Organizational Politics and Job Attitudes Relationship

**DOI:** 10.3389/fpsyg.2021.609842

**Published:** 2021-03-24

**Authors:** Hira Salah ud din Khan, Shakira Huma Siddiqui, Ma Zhiqiang, Hu Weijun, Li Mingxing

**Affiliations:** ^1^School of Management, Jiangsu University, Zhenjiang, China; ^2^Department of Applied Psychology, National University of Modern Languages (NUML), Islamabad, Pakistan; ^3^Air University School of Management (AUSOM), Air University, Islamabad, Pakistan; ^4^School of Archaeology, Jilin University, Changchun, China

**Keywords:** perceived organization politics, job attitudes (job satisfaction and job involvement), personal resources, political skill, work ethic

## Abstract

Drawing insight from affective events theory, this study presents a new dimension of perceived organizational politics and job attitudes. The motivation for this study was based on the fact that perceived organizational politics affect job attitudes and that personal resources (political skill and work ethic) moderate the direct relationship between perceived organizational politics and job attitudes in the context of the higher-education sector. In this regard, the data was collected through purposive sampling from 310 faculty members from higher-education institutions in Pakistan. To test the relationships among the variables, we employed structural equation modeling via the AMOS software version 24.0. The results indicated that perceived organizational politics were significantly negatively related to job satisfaction. Moreover, perceived organizational politics were non-significantly related to job involvement. Political skill and work ethic weakened the relationship between perceived organizational politics and job satisfaction. We anticipated that these personal resources could mitigate the negative effect of perceived organizational politics and job attitudes. This study also suggests organizations to train their employees to develop essential personal skills.

**“*The person who says I'm not political is in great danger, only the fittest will survive*,** ***and the fittest will be the ones who understand their office's politics” (Jean Hollands)***

## Introduction

Undoubtedly, employees will inevitably have perceptions about the context of the political environment in an organization (Hochwarter et al., 2020). These perceptions entail negative and positive outcomes, since perceptions permeate within the corridors of an organization (Asrar-ul-Haq et al., 2019). In light of this, perceived organizational politics (POP) have become a major issue for organizational researchers (Vigoda, 2002; Miller et al., 2008; Drory and Vigoda-gadot, 2010; Naseer et al., 2016). According to scholars, POP play a pivotal role in organizations, culminating in undesirable phenomena that hinder employee productivity (Franke and Foerstl, 2018; Lin and Sun, 2018; Crawford et al., 2019). Given this consideration, the growing body of literature appears to focus on perceived POP as a major job stressor that can potentially result in negative outcomes, not just for individuals but also for the organization itself (Maslyn et al., 2017; Webster et al., 2018; Guo et al., 2019; Landells and Albrecht, 2019). This particular aspect has threatened the effectiveness of the education sector by creating an unhealthy environment that affects the mental well-being of academic staff (Landells and Albrecht, 2015). Education is viewed from different angles, especially at higher levels, as a license for individual freedom and productivity in general (Li et al., 2018). Furthermore, scholars have expounded that the workforce in higher education is susceptible to politics in many ways, which adversely impacts their performance (Asrar-ul-Haq et al., 2019).

**Figure 1:**
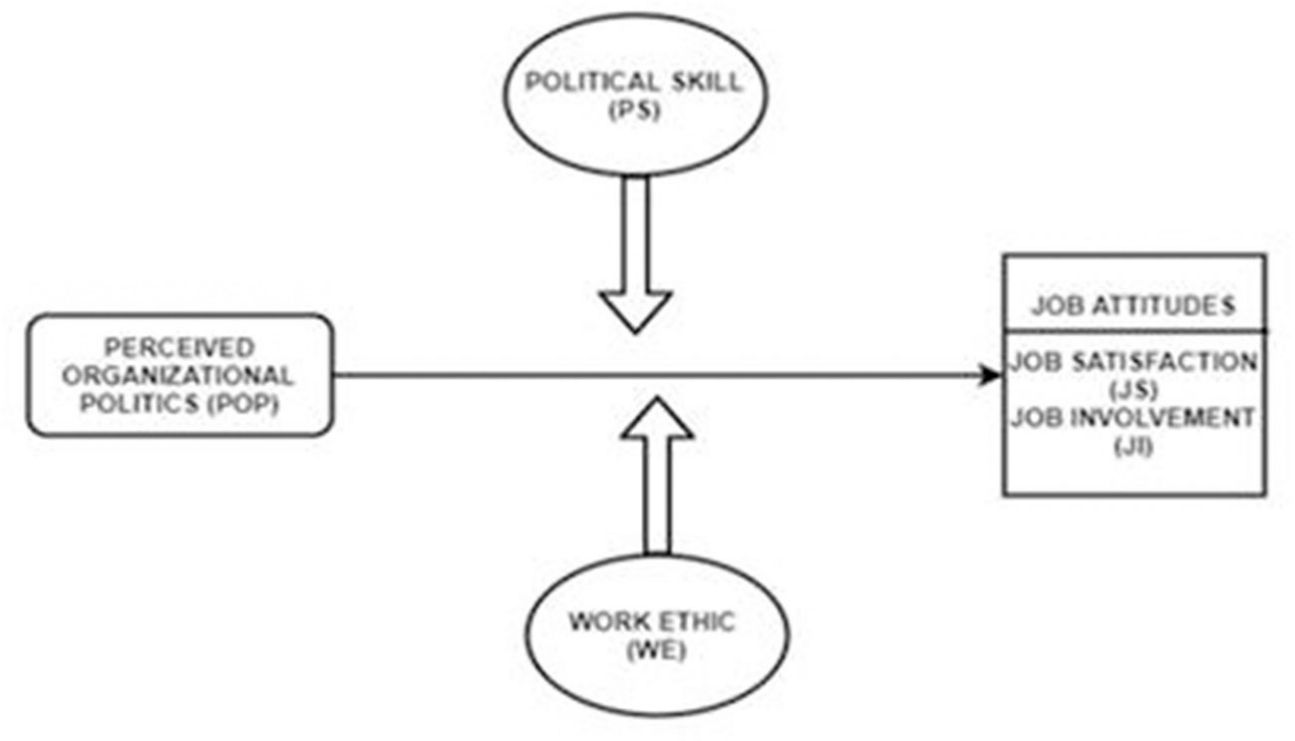
Conceptual framework.

Despite the negative aspects of POP, some hold the view that it is a panacea for employee growth (Bergeron and Thompson, 2020). Thus, scholars contend that POP can potentially trigger a desire in the workforce to protect the resources of their organization (Drory and Vigoda-gadot, 2010; Eldor, 2016; Ferris et al., 2017; Landells and Albrecht, 2019). Given this, scholars have elaborated that the workforce must be able to handle stress and strain in any kind of situation (Degbey and Einola, 2019). Employees with the potential to control their surroundings become the vanguard of leadership and champion others within an entity (McNeil et al., 2019). Based on this logic, individual personal resources are important factors that trigger motivation and inspiration in employees to combat the negative and undesired effects of politics (Landells and Albrecht, 2015). Thus, we contend that personal resources such as political skill (PS) and work ethic (WE) are key factors that help employees harness their coping mechanisms to minimize the negative effects of POP. In this context, PS are understood as a blend of positive attributes required to survive in present work settings and to promote employee participation in an organization, leading to job satisfaction (JS) and accomplishments (Eldor, 2016; Perrewé and Ferris, 2016). WE is explained as devotion and dedication toward work, ultimately resulting in organizational progress (Cao and Hill, 2019). Given this, Chen and Fellenz (2020) view personal resources as a coping mechanism through which individuals can gain positive energy to manage events that might jeopardize their progress. Similarly, personal resources have been found to moderate the detrimental effect of job stressors that enhance employees' motivation (Peng et al., 2010). Although the literature has paid considerable attention to job stressors (Landells and Albrecht, 2013; Jam et al., 2016; Maslyn et al., 2017), to the best of our knowledge, limited empirical studies have investigated the interaction effects of POP and personal resources (PS and WE) in the higher-education sector of Pakistan. Furthermore, the extant literature highlights the important linkage between emotions and how workforce assesses and reacts to their work atmosphere (Reina et al., 2018; Sewell, 2020). However, limited studies have investigated the interaction effects of POP and personal resources in the domain of higher education (Khan H. S. et al., 2019). Further, regarding the contextual dimension, prior studies mostly situate POP in Western culture, but this study focuses on the Asian region, particularly the educational sector of Pakistan. To address this knowledge gap, we built on affective events theory (AET) (Weiss and Cropanzano, 1996) and research focused on POP and job attitudes to develop a framework to enrich the literature in this field (see Figure 1).

In a quest to contribute to knowledge, the present study is followed by literature review on the study constructs, then on the methodology adopted to investigate the conceptual model. The next section comprises of the results, and the last section focuses on the discussions, conclusion, and future study.

## Literature Review

### Affective Events Theory (AET)

Recently, the notion of POP has captured the attention of scholars owing to its significance in organizational studies (Deale and Lee, 2016; Cho and Yang, 2017). In this regard, different perspectives have referred to this concept in the academic domain, particularly issues pertaining to organizations (Atta and Khan, 2016; Asrar-ul-Haq et al., 2019). Following this logic, a substantial number of scholars and a series of academic papers have highlighted POP through the lens of several theories (Lin and Sun, 2018; Crawford et al., 2019). A review of the literature indicated that AET has paid little attention to POP (Rosen et al., 2009); thus, the current study employs a survey of the POP literature through AET theory. This theory seeks to emphasize on the affective experiences (e.g., emotions and temperaments) that play a vital role in defining behaviors and attitudes (Miao et al., 2017). However, scholars have further explicated that significant resources within the means of employees will propel them to mitigate this issue (Abdullah et al., 2017; Bauwens and Decramer, 2019). This theory explains that the prevalence of adverse environments perceived by the workforce jeopardizes their positive performance and leads to more negative emotions such as anxiety and frustration (Tang et al., 2020).

Indeed, the burgeoning literature attests that when employees are in a politically intense environment, it will result in self-serving behaviors; thus, in this regard, their energy for other activities will be exhausted (Crawford et al., 2010). Additionally, when employees perceive that they face high stressful environments such as politicking and when things are not going favorably for them, they assume that all decisions made in the organization are against them (Bergeron and Thompson, 2020). Specifically, this study transfuses AET by viewing politics as a negative emotional event and personal resources (PS and WE) as positive emotional responses that are appraised as pertinent to employees' objectives, well-being, or values and create positive job attitudes in the organization. PS and WE serve as fundamental personal resources to tap positive energy and to enable employees to learn from adverse work situations. In line with this, AET demonstrates that toxic work conditions are disastrous when affective events are missing (Tang et al., 2020). Similarly, studies indicate that in the presence of positive personal resources, a hostile work atmosphere may be less marked as a result of any support or positive energy that might drive employees and enhance their motivation to work harder to increase organizational output (Van Mol et al., 2017; Gerpott et al., 2020). Based on this evidence, the present study addresses this overlooked link by examining the moderating effect of the two crucial resources that are embedded in the workforce's personal abilities, PS and WE, which significantly contribute to organizational prosperity.

### Perceived Organizational Politics and Job Satisfaction

It is interesting to note that individuals hold different opinions and perceptions regarding organizational politics, a facet of the social fabric (Lin and Sun, 2018). As argued by Atta and Khan (2016), employees in organizations view politics within the entity as a negative element; thus, it has been established that this element depicts negative characteristics, thereby defined as POP. In consideration of JS, a growing body of literature has pointed out that JS is essentially based on the degree to which individuals exhibit passion and enthusiasm for their occupation (Purpora and Blegen, 2015). The seminal work of Ferris et al. (1989) indicated that POP are a significant predictor of job outcomes. A previous study found that POP were negatively related to JS and certain other job outcomes (Drory, 1993). Some empirical studies demonstrated the insignificant association between POP and JS; for instance, Parker et al. (1995) demonstrated a non-significant link between POP and JS. However, the study revealed that the workforce was less occupied in innovative work when it perceived high politics in the workplace (Parker et al., 1995). Subsequently, Bozeman et al. (1996) identified the impact of POP on various job outcomes. Scholars identified that work stressors affect adversely the job outcomes of workforce (Sarfraz et al., 2019; Sarwar et al., 2019; Khan et al., 2020). Given this, the burgeoning literature on POP presents mixed findings related to POP and work-related outcomes (e.g., Ferris and Kacmar, 1992; Miller et al., 2008; Chang et al., 2009; Abbas et al., 2014). Some scholars have pointed out that individuals' job attitudes are greatly affected by high work stressors such as POP in the workplace (Hsieh et al., 2016; Labrague et al., 2017). Given this, AET expounds that negative emotional events such as POP and employees' psychological elements such as job attitudes (i.e., JS) are greatly affected. Based on this argument and theory, we hypothesize the following:

**H1:** POP is significantly and negatively related to JS.

### Perceived Organizational Politics and Job Involvement

Job Involvement (JI) can lead to positive psychological feelings that allow employees to concentrate on and prioritize the positive aspects of jobs (Lambert et al., 2018). By doing so, it is plausible to conclude that POP embody both negative and positive outcomes for employees and managers in an organization. In line with this, researchers exhort managers to consider any negative tendencies that might derail the productivity of the organization with respect to job attitudes (Gerpott et al., 2020). In this regard, an unfriendly environment in the workplace contributes to lower productivity coupled with low JI, participation, or unethical behaviors (Rosen et al., 2006; Rawwas et al., 2018; Shah et al., 2020), even though previous studies acknowledge both positive and negative effects associated with POP with respect to job consequences (Miller et al., 2008). Likewise, the literature concedes that there is a prevalence of some disastrous effects of POP that pervade across cultures (Tsui et al., 2007; Naseer et al., 2016). Given this, politics are considered to be a detrimental use of personal power to fulfill self-interests rather than organizational goals (Aggarwal et al., 2018). Subsequently, the workforce uses harmful power to gain personal benefits (Maslyn et al., 2017) which eventually leads to adverse outcomes (Franke and Foerstl, 2018). A growing body of literature has revealed that politics have a negative impact on individual and organizational performance (Landells and Albrecht, 2015; Malik et al., 2018; Rawwas et al., 2018). Against this backdrop, employees in a political environment are likely to reflect a low JI. Moreover, AET also supports the notion that in the case of stressful environments, workforce outcomes are affected, thus promoting negative job attitudes and diminishing the JI. Therefore, we hypothesize the following:

**H2:** POP is significantly and negatively related to JI.

### Political Skill as a Moderator in the Relationship Between Perceived Organizational Politics and Job Satisfaction

Political skill is defined as “the ability to effectively understand others at work, and to use such knowledge to influence others to act in ways that enhance one's personal and/or organizational objectives” (Ferris et al., 2005, p. 127). Extensive literature has demonstrated the vital role of PS in helping the workforce cope with stress and influence the minds of others (Brouer et al., 2011; Blickle et al., 2013; Zaman et al., 2019). Ferris et al. (2007) demonstrated that politically skilled employees also have the ability to adjust to every situation while maintaining legitimacy and openness (Crawford et al., 2019). This scenario motivates employees to value and appreciate politically skilled people as the embodiment of hope and reliance (Cullen et al., 2015). Moreover, Munyon et al. (2013) supported the notion that PS contributes immensely to reducing the hostile impact of POP, motivating employees to demonstrate a spirit of strong commitment to their respective tasks.

Additionally, the rich literature on PS asserts that employees facing challenges related to social power often manifest unsatisfactory behaviors and attitudes (Ferris et al., 2009). Crawford et al. (2019) argued that when politically skilled people encounter stressors in the workplace, the stressors ultimately produce less negative impacts on employees' job attitudes. From the lens of AET, personal resources are defined as positive emotional, societal, physical, or organizational indicators of a job. These indicators aid in the realization of work objectives that serve as catalysts in minimizing negative events and related emotional and physiological costs. Previous studies have also evidenced that personal resources fuel personal growth and development (Zhang et al., 2018; De Clercq and Belausteguigoitia, 2020). Therefore, we consider PS to be one of the personal resources for mitigating the adverse impact of POP on job attitudes such as JS. Based on this discussion and theory, we hypothesize the following:

**H3:** PS moderates the relationship between POP and JS such that the relationship will be weaker when PS is high.

### Political Skill as a Moderator of the Relationship Between Perceived Organizational Politics and Job Involvement

As established in the literature, individuals who are endowed with PS play an instrumental role in getting along with employees, who must endure this advantage over them (Perrewé et al., 2004; Butt et al., 2017; García-chas et al., 2018). These individuals with PS easily adjust to any political environment and efficiently employ social skills in motivating subordinates or employees in an organization to boost their JS (Kapoutsis et al., 2016; Cong et al., 2017). In addition, PS helps employees to communicate barriers and thus minimizes the stress resulting from ambiguity (Crawford et al., 2019). Specifically, politically skilled individuals are comfortable in their work environment, which promotes their work outcomes (Khan et al., 2018). By virtue of their potential network, they can easily gather information pertaining to the organization, which they can rely upon to either improve or amend certain conditions (Perrewé et al., 2004; Ferris et al., 2007; Cullen et al., 2015); for instance, a high PS level is related to greater work rewards for employees who exhibit good behavior in the organization. In this regard, those with PS are more able to win the favor of the organization than those who lack PS (Khan et al., 2018). Consequently, the hopes of individuals with PS are likely to rise, thus eliminating the negative consequences that may hinder the progress of POP and JI. This, in turn, suggests that with the prevalence of stressors in an organization such as POP, individuals with PS tend to have maximum control over all kinds of difficulty (Kimura, 2014). Accordingly, AET theory indicates that positive emotion events such as personal resources moderate the link between negative factors and exhaustion in the midst of difficult work conditions faced by employees; as a result, they experience fewer negative feelings (Farid et al., 2019). These attributes of personal security (i.e., self-confidence) in the context of the political environment (Ferris et al., 2007) will result in an increase in the political acumen of individuals' PS in the organization, which will lead to lower POP. Therefore, based on the theory and arguments advanced above, we propose the following:

**H4:** PS moderates the relationship between POP and JI such that the relationship will be weaker when PS is high.

### Work Ethic as a Moderator in the Relationship Between Perceived Organizational Politics and Job Satisfaction

Work ethic has become a focal subject of many scholarly studies (Osibanjo et al., 2015; Meriac and Gorman, 2016; Grabowski et al., 2019). For instance, Rawwas et al. (2018) identified WE as a vital personal attribute that speaks volumes about employees' recognition even in the absence of organizational control. Meriac et al. (2010) stated that WE characterize the principal importance of work in one's attitude and convictions. Against this background, a stream of scholars has argued that WE may not be static but can be viewed as dynamic. As a result, employees with a high WE exhibit positive job attitudes that embody JS and moral standards (Osibanjo et al., 2015). Work ethic has been assumed to be a key factor that affects an individual's feelings and that somehow promotes JS in a given environmental setting, propelling the positive attitudes of employees (Mauno et al., 2016). Given this, we concur that the positive job evaluations of employees may trigger their zeal to work as a team, which may ultimately lead to higher productivity and personal satisfaction (Athar et al., 2016; Gheitani et al., 2018). This implies that it would behoove managers to ensure that adequate and appropriate measures are put in place to motivate employees to do their best at whatever tasks assigned to them. Thus, an enabling environment must be created by leaders to propel employees' JS (Komari and Djafar, 2013; Fakunmoju, 2018; Taufail et al., 2018). As argued by researchers, AET plays an indispensable role via personal resources that aid in addressing the employee stress caused by tedious and high stressful environment, such as POP (De Clercq and Belausteguigoitia, 2020). Some have argued that WE serves as an efficient and important instrument capable of reducing the volume of employee stress in the midst of adverse work conditions (Grabowski et al., 2019). In line with this logic, the substantial literature on POP attests that the Islamic WE plays a moderating role in stressful work environments characterized by POP and unethical leadership styles (Rawwas et al., 2018; Raja et al., 2019; Smirnova et al., 2019). Nonetheless, it is still unclear how WE can ameliorate stressful environments characterized by POP. It is therefore imperative to infer that WE play a moderating role in the association between POP and JS. Hence, we postulate the following:

**H5:** WE moderates the relationship between POP and JS such that the relationship will be weaker when WE is high.

### Work Ethic as a Moderator in the Relationship Between Perceived Organizational Politics and Job Involvement

As established by extant studies, WE has a pivotal role in high levels of self-motivation and initiative taking (Baumann et al., 2016; Chasovschi, 2016; Muenjohn and Mcmurray, 2017). Thus, from a practical lens, a robust WE epitomizes task completion at the right time (Mauno et al., 2016; Meriac and Gorman, 2016). The underlining assumption of this logic is that the essence of WE is the appreciation of needs that can mutually benefit both the workforce and employers (Van Ness et al., 2010). Based on this, Schrift et al. (2016) and Miñon (2017) argued that individuals with high WE appear to have a high moral motivation that triggers their ethical commitment to be involved in their jobs and may give them a tendency to fit into systems. Previous works on Islamic WE have demonstrated its moderating effects on POP and found that employees with an Islamic WE demonstrate the characteristics of respect, generosity, commitment, fairness, patience, teamwork, and work devotion (Khan H. S. et al., 2019). Thus, this strengthens employees' ability to resist any form of negative politics, which consequently translates into high JI (Mauno et al., 2016; Meriac and Gorman, 2016). In a similar vein, a growing body of literature has documented that the Islamic WE is a vehicle for positive job outcomes such as fairness, work devotion, and ethical means of acquiring income (Khan et al., 2013; Rawwas et al., 2018). The debates on POP have generated several opinions among scholars on the basis that contextual factors or personal resources have a significant link with stressful environments characterized by POP. In other words, they are essential variables that might influence the association between POP and job outcomes (Hochwarter et al., 2020). Therefore, this study further extends the POP literature by considering the moderating role of WE in the association between POP and attitudes. Based on the above propositions espoused by scholars, the following is proposed:

**H6:** WE moderates the relationship between POP and JI such that the relationship will be weaker when WE is high.

## Methods

### Participants and Procedures

To understand the impact of POP at workplace, we conducted this study on faculty members of the higher-education sector of Pakistan. Particularly, this research focused on teachers' underlying severe stress caused by POP which is a great challenge that affects not only the progress of the higher educational institutes but also students' productivity (Anjum et al., 2018; Kachhawa and Gajraj, 2018; Asrar-ul-Haq et al., 2019). Therefore, we targeted faculty members from higher educational institutes of Pakistan.

Prior to the survey, we distributed 25 questionnaire to respondents and 5 to experts to meet the requirement of the pilot study. Having observed the pilot study requirement, we then collected data for our quantitative study. The respondents were briefed about the objectives of the study, and the ethical code of anonymity of the people and workplace was ensured. We collected data via self-reported questionnaire from selected universities of diverse regions by purposive sampling technique. This method was adopted in order to approach a specific group of people as they fit well in the framework of the survey (Tongco, 2007; Etikan, 2016; Serra et al., 2018). In line with this, previous studies indicate that a threshold of sample size 30–500 is adequate for conducting the survey (Roscoe, 1975). Therefore, this investigation gathered data from 310 faculty members of universities by employing purposive sampling technique in accordance with studies of Bodla et al. (2015), Khan and Hussain (2016), and Ahmed et al. (2018). After seeking permission from the respective universities, the questionnaires were distributed to the participants in person and via email. The survey questionnaire which was not properly filled in was discarded. Remaining questionnaires out of 400 which totaled 310 with a response rate of 88% constituted the final data for study analysis.

### Demographic Information

The hypotheses were therefore tested with a final sample of 310 participants consisting of 152 men (49%) and 158 women (51%). The age of employees ranged from 21 to 60 years (*M* = 1.70, *SD* = 0.84). Workforce tenure in the present job ranged from <1 to 25 years (*M* = 1.41, *SD* = 0.83). Employees' tenure in the previous workplace ranged from <1 to 25 years (*M* = 1.60, *SD* = 1.02). 26% (*n* = 81) respondents had master's degree whereas 50% (*n* = 157) were MS/MPhil and 17% (*n* = 50) were Ph.D. Married faculty was 55% (*n* = 171), 42.9% (*n* = 133) were unmarried, 1.1% (*n* = 4) were divorced, and 1% (*n* = 2) were widow. The data was collected from public (*n* = 147, 47.4%), and private (*n* = 163, 52.6%) universities of Pakistan.

### Measures

To meet the methodological requirements for any given scientific research suggested by the experts (Basar and Basim, 2016; Erkutlu and Chafra, 2016), we adopted and modified a five-point Likert scale ranging from 1 = strongly disagree to 5 = strongly agree. The items were coded such that high values represented high levels of each construct.

#### Perceptions of Organizational Politics Scale (POPS)

We quantified POP through a 12-item scale sourced from Ferris and Kacmar (1992). Items found on the 12-item scale included the following: “Favoritism rather than merit determines who gets ahead around here.” Additionally, items 6, 7, and 11 were negatively keyed and were reverse coded.

#### Job Satisfaction

JS was analyzed using a four-item scale, consistent with Churchill et al. (1974) and Hackman and Oldham (1975). The following is an example item included in the JS scale: “All in all, I am satisfied with the people in my work group.” Scale item 3 was negatively keyed and reverse coded.

#### Job Involvement

Here, we quantified JI using six items, in line with the logic of Lodahl and Kejnar (1965). To ensure vivid comprehension of this construct, the following is an example of a sample questionnaire item: “The major satisfaction in my life comes from my work.” Scale item 6 was negatively keyed and reverse coded.

#### Political Skill

In this construct, we further quantified PS by means of the scaling procedure of Vigoda-gadot and Meisler (2010). The items in this scale were vigorously analyzed and reduced to eight items, in line with Ferris et al. (2005). The following is a typical example of a sample item in the questionnaire: “I always seem to instinctively know the right thing to say or do to influence others.”

#### Work Ethic

This construct was composed of 26 items and was quantified though the scale by Rosseel (1985). To paint a clearer picture of this construct, the following is an example of a sample questionnaire item: “Work is the most important thing in life.”

#### Control Variables

Demographic variables such as gender, age, education, marital status, and job tenure affected the relationship between the variables in the current study. Previous empirical studies have shown that these variables account for variance in this relationship (Allen and Meyer, 1993; Ferris et al., 2002; Clerq et al., 2017). Therefore, these demographic variables were controlled statistically while testing the hypotheses.

## Results

### Analysis Strategy

To ascertain the association among the study measures, we employed SPSS version 22 to analyze the descriptive statistics. In this factor, the models of each measure were examined through confirmatory factor analyses using AMOS software 24.0. Therefore, the direct and moderating tests were executed through structural equation models using this software. The links between the latent and observed constructs were measured via structural models. The study variables depicted the latent and observed variables. For instance, this study utilized POP as a latent variable that consisted of 12 items. Likewise, JS, JI, PS, and WE were also considered as latent variables.

### Results of the Confirmatory Factor Analysis

To validate the measurement model of the study variables, confirmatory factor analysis was employed. It produced a structural relationship between the latent variables. The measurement model was comprised of 5 constructs (POP, JS, JI, PS, and WE) and 47 observed variables. Table 1 shows the factor loadings, average variance extracted, and construct reliability of the scale. If any item had a factor loading of ≥0.40 (Cua et al., 2001), it was included for further analysis. An AVE value of >0.50 was accepted for adequate convergent validity of the scale. A CR value of 0.70 or higher was accepted for good reliability. Any observed variables of the latent construct that showed suboptimal values were dropped for further analysis. This shows that the latent indicators showed a significant link, which indicates that their factor loadings were above 0.30 and met the minimum threshold value (Hair et al., 2006), as cited in Mahembe and Engelbrecht (2013).

**Table 1:** Convergent validity: factor loadings, average variance extracted (AVE), and construct reliability of scale.

**Name of variable**	**Items**	**Factor loading**	**AVE score**	**CR values**
POP			0.59	0.94
	POP1	0.58		
	POP2	0.60		
	POP3	0.58		
	POP4	0.83		
	POP5	0.87		
	POP6	0.89		
	POP7	0.90		
	POP8	0.79		
	POP9	0.80		
	POP10	0.81		
	POP11	0.79		
	POP12	0.68		
JS			0.73	0.91
	JS1	0.87		
	JS2	0.86		
	JS3	0.83		
	JS4	0.85		
JI			0.56	0.88
	JI1	0.84		
	JI2	0.84		
	JI3	0.83		
	JI4	0.84		
	JI5	0.52		
	JI6	0.52		
PS			0.64	0.92
	PS1	0.67		
	PS2	0.64		
	PS3	0.69		
	PS4	0.89		
	PS5	0.86		
	PS6	0.90		
	PS7	0.88		
WE			0.54	0.95
	WE1	0.61		
	WE2	0.59		
	WE3	0.66		
	WE4	0.87		
	WE6	0.85		
	WE7	0.85		
	WE9	0.86		
	WE10	0.77		
	WE11	0.78		
	WE12	0.59		
	WE14	0.78		
	WE15	0.72		
	WE19	0.67		
	WE20	0.69		
	WE21	0.65		
	WE22	0.70		
	WE23	0.83		
	WE26	0.62		

The results showed that all of the loading values of POP, JS, and JI were within the acceptable range, but one item from PS and eight items of WE were removed due to poor loading values at the first level of the CFA. After removing all poor items, as Table 1 shows, the factor loading, AVE, and CR values were within the acceptable range.

### Discriminant Validities of the Study Variables

The discriminant validities of the present study measures were obtained by conducting a master validity test in AMOS (Gaskin and Lim, 2016), as shown in Table 2. The results of the correlational matrix showed the link between POP and job attitudes (JS and JI), as well as between personal resources such as PS and WE moderator variables with job attitudes. It was found that POP had a significant negative association with JS but an insignificant one with JI. PS and WE were positively related with all of the other study constructs. The study constructs indicated adequate internal consistency (Cronbach's alpha and composite reliability >0.60). The square root of the average variance extracted for each measure was higher than the correlations among them and with other latent constructs representing discriminant validity (Fornell and Larcker, 1981). We assessed the convergent validity and discriminant validities by examining the loadings of the measurement items on the reflective construct and found acceptable results (Hu et al., 1999).

**Table 2:** Mean standard deviations, correlations, and reliabilities of all the study variables.

**Study variables**	**Mean**	***SD***	**α**	**1**	**2**	**3**	**4**	**5**
1. POP	3.08	0.63	0.94	(0.77)				
2. JS	2.90	0.59	0.91	−0.360^**^	(0.85)			
3. JI	3.19	0.37	0.88	0.058	0.167^*^	(0.75)		
4. PS	3.07	0.61	0.92	0.194^**^	0.202^**^	0.125^*^	(0.73)	
5. WE	3.04	0.59	0.95	0.204^**^	0.117^*^	0.117^*^	0.252^**^	(0.80)

** p < 0.01 and

* *p < 0.05*.

*POP, perceived organizational politics; JS, job satisfaction; JI, job involvement; PS, political skill; SD, standard deviation*.

*Square of average variance explained (AVE) values are shown in parenthesis*.

### Model Fit Analysis

According to scholars, SEM is a multifaceted methodological tool embedded in many programs such as AMOS. In view of this, we employed AMOS to evaluate the fit of the anticipated model in line with Khine (2013). Similar studies have also employed AMOS in the analysis of relationships that exist between POP, job attitudes, and personal resources. A model fit analysis of the study variables was conducted by model fit analysis in AMOS (Gaskin and Lim, 2016), as shown in Table 3. Items with low loadings were discarded to allow the authors to meet the objective of model fitness. The results in Table 3 indicated the values of model fit where chisq/*df* = 2.77 which is <3 as recommended by Hu et al. (1999) and Hair et al. (2010). Moreover, values of Goodness of Fit Index (GFI) = 0.98, Adjusted Goodness of Fit Index (AGFI) = 0.85, Comparative Fit Index (CFI) = 0.94, Trucker–Lewis Index (TLI) = 0.91, and Normative Fit Index (NFI) = 0.94 also meet the threshold criteria suggested above 0.90 (Hu et al., 1999; Hair et al., 2010, 2014a,b), and values of Root Mean Square Residual (RMR) = 0.03 and Root Mean Square Error of Approximation (RMSEA) = 0.04 also confirmed a good model fit as the values are <0.080 (Hu et al., 1999; Hair et al., 2010). Thus, the results indicated that all model fit indices showed values above the threshold criteria (Hair et al., 2014a,b). This shows that the model had a very good fit with the data.

**Table 3:** Model fit measure.

**Model fit criteria**	**Measurement model**	**Acceptable range**
CMIN	853	–
DF	305	–
CMIN/DF	2.77	1–3
GFI	0.986	>0.90
AGFI	0.857	>0.80
CFI	0.943	>0.95
TLI	0.910	>0.90
NFI	0.947	>0.90
RMR	0.037	<0.09
RMSEA	0.041	<0.08

### Common Method Bias (CMB)

In order to test a scale's CMB, researchers test the scale to measure the variance of the whole scale. CMB occurs when variations in responses are caused by the instrument rather than the actual predispositions of the respondents that the instrument is intended to uncover. The results in Table 4 show that there was no issue with CMB in the scale used in this study, with a cumulative variance of 47 items (28.92%), which is <50% (Gaskin and Happell, 2014).

**Table 4:** Total variance explained.

**Component**	**Initial eigenvalues**			**Extraction sums of squared loadings**		
	**Total**	**% of variance**	**Cumulative %**	**Total**	**% of variance**	**Cumulative %**
1	13.591	28.916	28.916	13.591	28.916	28.916
2	7.104	15.115	44.031			
3	5.099	10.848	54.879			
Up to 47	0.095	0.202	100.000			

### Path Analysis via Structural Equation Modeling

The theoretical framework was split into three models: that is, in the first model, the impact of POP was tested on JS and JI. In the second model, the impact of POP and the moderating variable PS was tested on JS and JI. In the third model, the impact of POP was tested on JS and JI using WE as a moderating variable. Before testing the SEM in AMOS, the regression assumptions were tested using SPSS. The data had no normality or validity issues, and the accuracy of the results depended on the validity of all assumptions of the regression analysis (Chatterjee and Hadi, 2015).

### Descriptive Statistics and Correlations

Table 2 illustrate the values of descriptive statistics, reliability, and correlations, where the reliability statistics of all study variables are above the minimum acceptance criteria 0.700 as suggested by van Griethuijsen et al. (2014). Moreover, bivariate correlational values in the table shows that POP negatively and significantly correlated with JS (*r* = −0.360, ^*^P < 0.05); moreover, POP positively and significantly correlated with PS (*r* = 0.194, ^**^p < 0.01) and with WE (*r* = 0.204, ^**^p < 0.01), but there was no significant correlation found between POP and JI (*r* = 0.058, n.s). Furthermore, JS was found to be positively and significantly correlated with JI (*r* = 0.167, ^*^p < 0.05), with PS (*r* = 0.202, ^**^p < 0.01), and with WE (*r* = 0.117, ^*^p < 0.05). Additionally, JI was also found to be significantly and positively correlated with PS (*r* = 0.125, ^*^p < 0.05) and with WE (*r* = 0.117, ^*^p < 0.05); lastly, PS was positively and significantly correlated with WE (*r* = 0.252, ^**^p < 0.01).

#### Model 1: Direct Relationship

**Figure 2:**
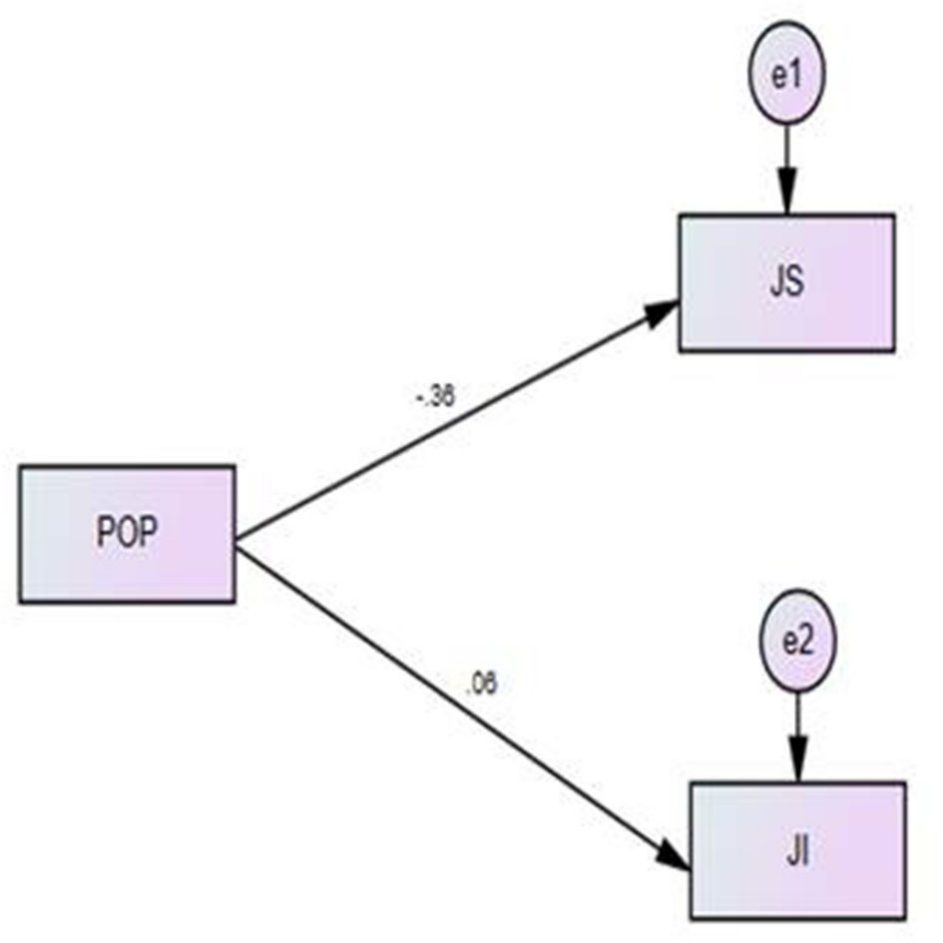
Structural Model for direct impact of POP on JS and JI.

Figure 2 and Table 5 show the results produced by the AMOS software. The analysis was performed using the maximum likelihood method, which is the default function of this software. The estimate (beta) value is reported to test the impact of each variable impact on the dependent variable. The critical ratio (CR), which is also denoted as the *t*-value, is also presented in the table. The CR value is acceptable if it is > ±1.96 (95% level of confidence). In the table, the *p*-value is reported as <0.01 or <0.05 for the significance of the relationship.

**Table 5:** Regression weights.

**IV**		**DV**	**Estimate**	**C.R**.	***P***	**LLCI**	**ULCI**
POP	→	JS	−0.360	−6.791	0.000	−0.479	−0.165
POP	→	JI	0.058	1.019	0.308	−0.078	0.150

*IV, independent variable; DV, dependent variable; C.R, critical ratio; LLCI, lower level of confidence interval; ULCL, upper level of confidence interval*.

Table 5 displays the SEM results, showing that POP had a negative and significant impact on JS (beta = −0.360, *p* < 0.001); the estimated value of −0.36 shows that if one unit increased in POP, it would have a 36% negative impact on JS. POP had an insignificant impact on JI (beta = 0.058, *p* > 0.05); thus, for moderation testing, we excluded JI as a dependent variable because POP had no direct impact on it, as explained by Cohen et al. (2003) and Hair et al. (2016). Table 5 shows the results for POP's effect on JS and JI.

#### Model 2: Moderation Analysis (PS as a Moderator)

**Figure 3:**
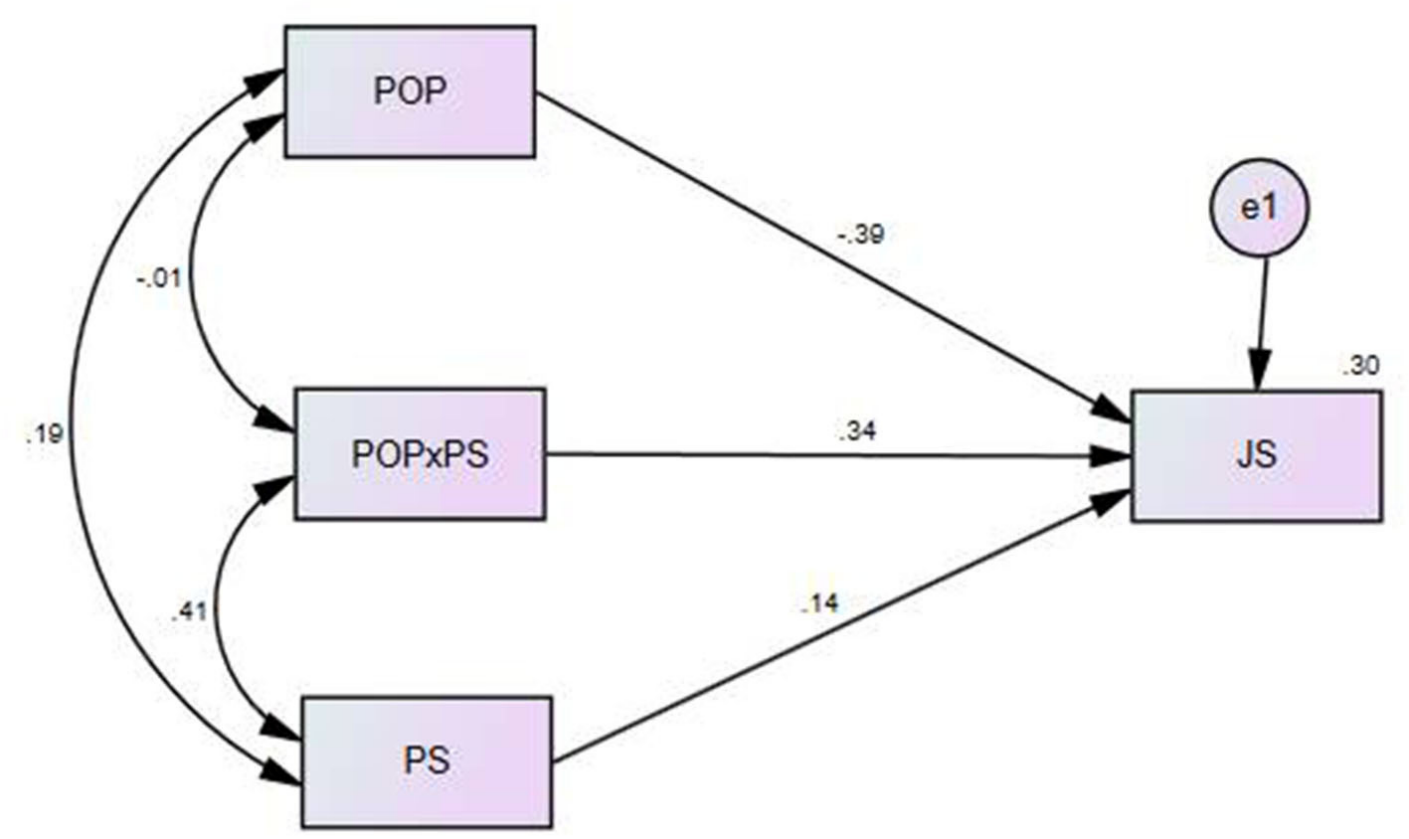
Structural Model for moderating impact of Political Skill.

In this study, PS was used as a moderator, POP as an independent variable, and JS as the dependent variable to test the moderation effect. Figure 3 shows the results of the moderation analysis using AMOS software.

Table 6 shows that POP had a significant impact on JS (coefficient = −0.386, *p* < 0.001), PS had a significant impact on JS (coefficient = 0.140, *p* < 0.01), and the moderating effect (POP × PS) also had a significant impact on JS (coefficient = 0.336, *p* < 0.001). The results showed that the moderating effect (interaction term) had a significant impact on JS.

**Table 6:** Regression weights: moderating effects of political skill.

**IV**		**DV**	**Estimate**	**C.R**.	***P***	**LLCI**	**ULCI**
POP	→	JS	−0.386	−7.908	0.000	−0.488	−0.197
PS	→	JS	0.140	2.616	0.009	−0.050	0.321
POP × PS		JS	0.336	6.409	0.000	0.064	0.142

*IV, independent variable; DV, dependent variable; C.R, critical ratio; LLCI, lower level of confidence interval; ULCL, upper level of confidence interval*.

#### Model 3: Moderation Analysis (WE as a Moderator)

**Figure 4:**
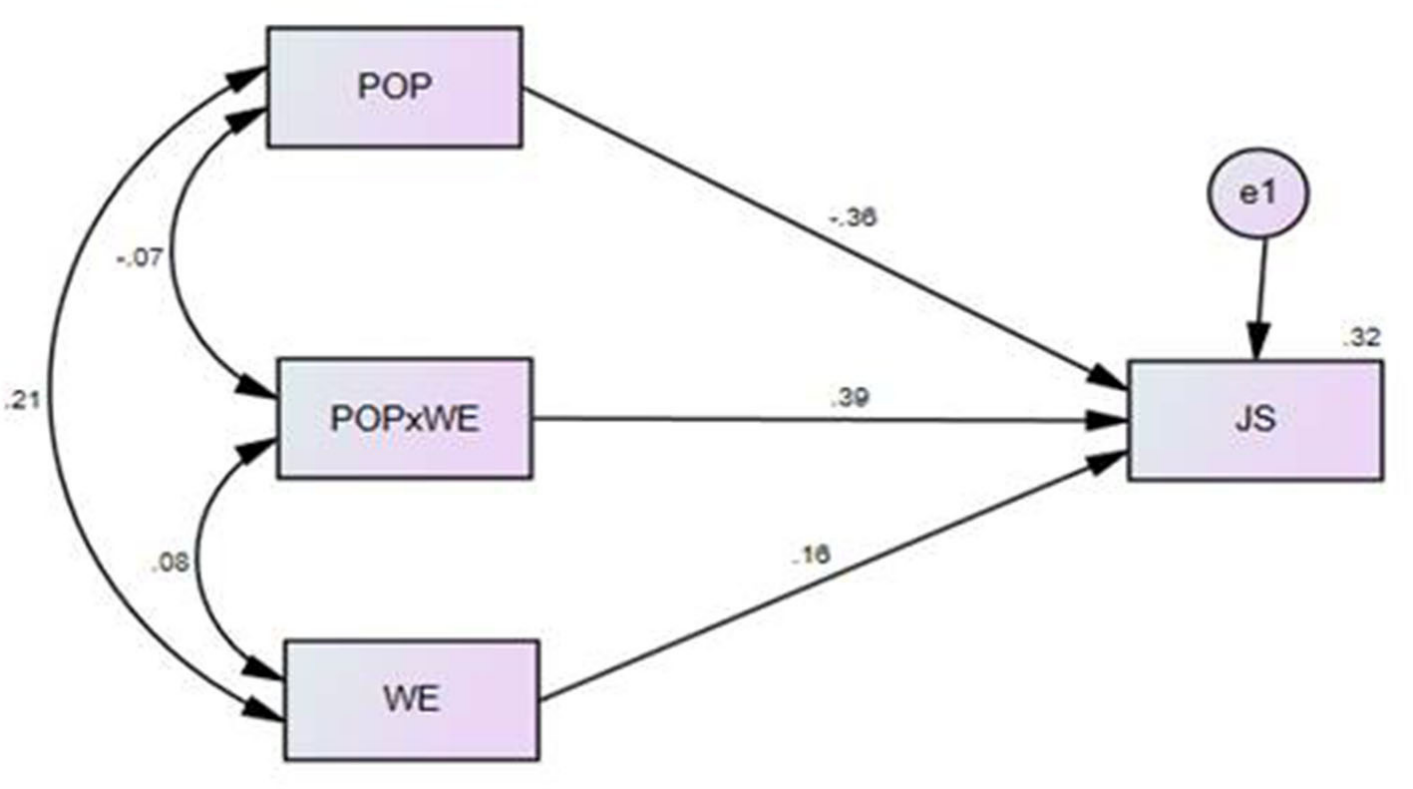
Structural Model for moderating impact of Work Ethic.

In this study, WE was investigated as a moderator, POP as an independent variable, and JS as a dependent variable to test the moderation effect. Figure 4 shows the results of the moderation analysis using AMOS software.

Table 7 shows that POP had a significant impact on JS (coefficient = −0.364, *p* < 0.001), WE had a significant impact on JS (coefficient = 0.157, *p* < 0.01), and the moderating effect (POP × WE) also had a significant impact on JS (coefficient = 0.393, *p* < 0.001). The results indicated that the moderating effect (interaction term) had a significant impact on JS.

**Table 7:** Regression weights: moderating effects of work ethic.

**IV**		**DV**	**Estimate**	**C.R**.	***P***	**LLCI**	**ULCI**
POP	→	JS	−0.364	−7.565	0.000	−0.478	−0.172
WE	→	JS	0.157	3.262	0.001	0.006	0.306
POP × WE		JS	0.393	8.306	0.000	0.080	0.166

*IV, independent variable; DV, dependent variable; C.R, critical ratio; LLCI, lower level of confidence interval; ULCL, upper level of confidence interval*.

**Figure 5:**
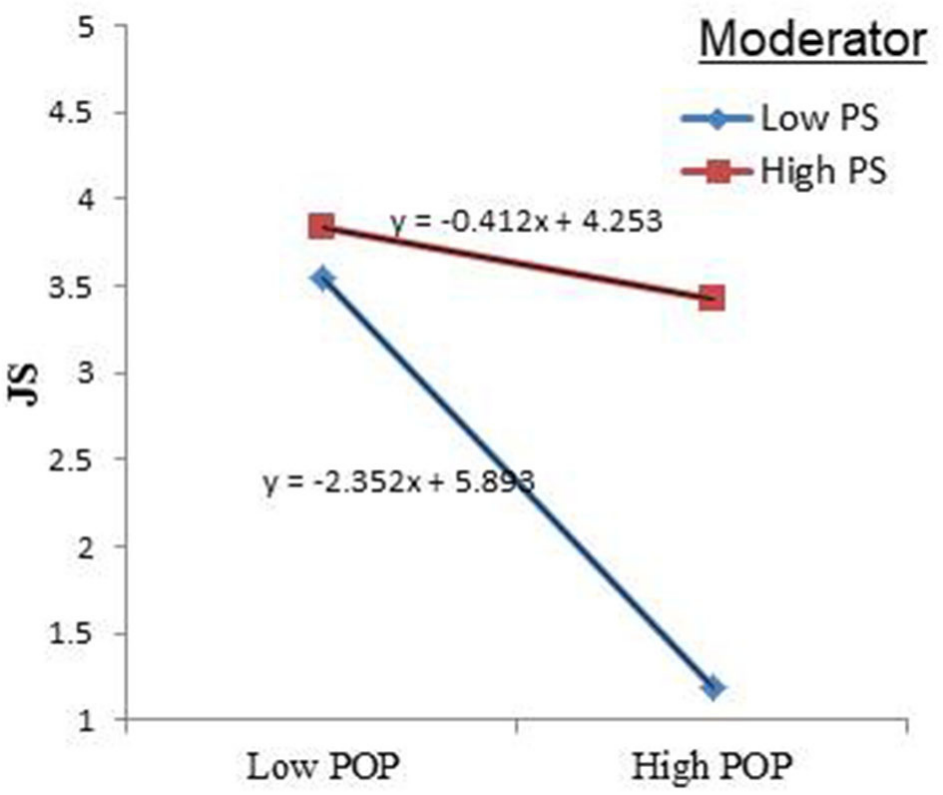
Interaction effect of Political Skill.

PS as a moderator also showed that the link between POP and JS was negative for the lines, as indicated by their negative slope (see Figure 5). Both the upper and lower lines had negative slopes. The upper line, which demonstrated a high level of the moderator variable PS, had a flatter slope, while the lower line, which indicated a low level of the moderator variable PS, had a steeper slope, with the interaction effect being positive. The slope of the high level of the moderator PS was −0.412, while the slope of the low level of the moderator PS was −2.352. Hence, the simple slope plot (Figure 5) supported the hypothesis of a positive interaction term: higher PS levels entail a weaker relationship with a high POP, resulting in a decrease in JS, while lower levels of PS lead to a reduction in JS as POP increases. Thus, higher the PS, the weaker the effect of POP on JS.

**Figure 6:**
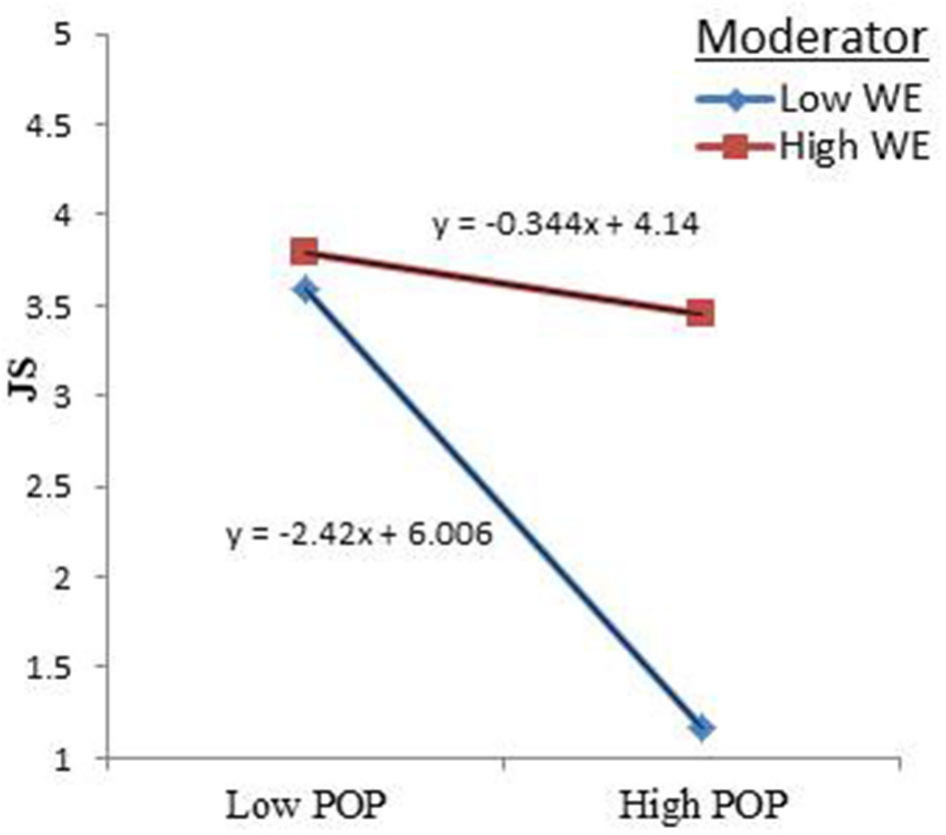
Interaction effect of Work Ethic.

Similarly, the slope plots, as shown in Figure 6, indicated that the relationship between POP and JS was the same, as the lines showed with their negative slopes. Hence, a higher level of POP resulted in a high level of JS. The slope of the high level of the moderator (WE) was −0.344, while the slope of the low level of the moderator (WE) was −2.42. Hence, the simple slope plot (Figure 6) supported the hypothesis of a positive interaction term: higher WE levels entail a stronger relationship with a high POP increasing JS, while lower levels of WE lead to a reduction in JS as POP increases. Thus, the higher the WE strategies, the weaker the negative effect of POP and JS.

Table 8 shows that the interaction term's *f*^2^ effect size for POP× PS had a value of 0.428, which indicated a large effect consistent with Aguinis et al. (2016). Overall, these results provide clear support for the notion that PS weakens the adverse association between POP and JS with a higher effect. Moreover, the interaction term's *f*^2^ effect size for POP × WE had a value of 0.471, which indicated a large effect consistent with Aguinis et al. (2016). Overall, these results provide clear support for the notion that WE weakens the adverse association between POP and JS. This suggests that the higher the WE, the weaker the adverse impact of POP and JS.

**Table 8:** *f*^2^ effect size of the moderating role of political skill and work ethic.

**Interaction effect**	**Coefficient**	**f^2^**	**Remark**
POP^*^ PS	0.336	0.428	Large
POP^*^ WE	0.393	0.471	Large

*Small: 0.0< f^2^ effect size <0.15; Medium: 0.15 < f^2^ effect size <0.35; Large: f^2^ effect size >0.35*.

## Discussion

Although a growing body of research has devoted considerable attention to the association between POP and Job outcomes in organizational settings, the extant literature has overlooked the moderating impact of personal resources such as PS and WE in the relationship between POP and job attitudes. To enhance the understanding of this topic, the present study fills the gap in the existing body of knowledge in the domain of organizational behavior by applying affective events theory to the higher-education sector of a developing country, Pakistan.

This study investigated POP and job attitudes through the lens of the workforce in the higher-education sector of Pakistan. In light of this, our findings revealed a direct negative link between POP and JS, in line with the proposition espoused by scholars that POP exerts some harmful effects on work attitudes and behavior (Khan et al., 2017; Hochwarter et al., 2020; Park and Lee, 2020). These negative harmful effects are the result of self-centered attitudes in the organization, which have the tendency to erode or diminish employees' energy levels, consistent with the findings of Ferris and Kacmar (1992) and Crawford et al. (2010). With regard to the findings of impact of POP, we found surprisingly the positive impact of POP on JI instead of negative and thus this hypothesis was not supported. A possible reason for this is that employees with a high perception of politics are likely to exhibit a high level of JI, as argued by Ferris and Kacmar (1992). Further, it is observed that employees when perceiving a high level of politics in the organizations tend to get more involved in their work so as to avoid the negative environment (Atta and Khan, 2016). Based on this logic, we suggest that to avoid politics, employees tend to overly engage in organizational tasks.

The results regarding the moderating effect of PS indicated a significant role in weakening the link between POP and JS. This stance was corroborated by Ferris et al. (2009) and Nikoletta and Petros (2015), who showed that employees with high PS encounter stress more easily because they are quick to adjust to social upheavals. Furthermore, they utilize any benefits that underpin POP for their personal growth and development (Crawford et al., 2019). As established by prior studies, employees who are endowed with high PS exhibit absolute JS (Kacmar et al., 2013; Rosen and Levy, 2013). Thus, when PS is high, the negative link between POP and JS, or any factors that will impede their productivity, is eliminated.

The empirical findings of the moderating impact of WE revealed the dampening effect of WE on the adverse effect of POP on JS. Through this personal resource, the workforce can attain a level of confidence that assists in realizing the assigned job, regardless of negative political strategies (Bentley et al., 2017; Naseer et al., 2018). In view of this, they have adequate energy to carry out extra work tasks that increase organizational efficiency. Studies on the moderators of the POP–job attitude association have revealed that the adverse effects of POP can be lessened by employees' skills or attributes (Kapoutsis et al., 2016; Bentley et al., 2017). Our finding on the moderating role of WE extends previous scholarly works that proposed the need for promising organizational features, such as WE, as a driver to translate into positive job attitudes. In this regard, employees with a high degree of WE are bestowed with high JS that translates into a healthy political atmosphere. Overall, the significant moderating role of personal resources established by this study is consistent with JD-R theory, which specifies that a hostile organizational work atmosphere diminishes positive job attitudes. On the other hand, the availability of resources leads employees to ignore negative tendencies that might emerge as a result of stressors (De Clercq and Belausteguigoitia, 2020). Thus, it is found that positive job attributes such as PS and WE can be beneficial for an organization.

### Theoretical Implications

This study adds to the literature and extends the research stream on job attitudes. First, our results enhance the literature of political perceptions by transfusing the AET through moderating mechanisms in the POP and job attitudes. The association between POP and job attitudes, as represented in this study, has essential implications for scholars' effort to comprehend the effects of POP on organizational outcomes (Maslyn et al., 2017; Landells and Albrecht, 2019). Some stream of scholars identified a negative relationship between POP and job attitudes by different theories (Atta and Khan, 2016; Cho and Yang, 2017; Khan N. A. et al., 2019). However, the present POP model has not been addressed through the lens of AET theory. We revealed that a job stressor such as POP represents as a negative emotional event in the work atmosphere that employees perceive it to be an obstacle and challenging in achieving their goals. Scholars have indicated that job attitudes play an essential role in enhancing the organizational performance (Park and Lee, 2020). Given this, our study indicated that an undesirable emotion event in the form of politics diminishes its positive impact.

Furthermore, the study revealed the essential personal resources such as PS and WE which serve as a moderating variable to reduce the detrimental effects of POP and that leads to positive job attitudes. Yet, extensive studies have revealed that POP is negatively linked to job outcomes (Cho and Yang, 2017; Malik et al., 2018; Asrar-ul-Haq et al., 2019). Scholars have demonstrated that job attitudes have a positive impact on organizational success (Park and Lee, 2020). This is contingent upon the fact that there are some motivating factors prevailing and are sustained by environmental stimuli like pay increases and self-recognition (Sen et al., 2018), which results in positive job attitudes. Our findings are consistent with the assumption of AET which showed that the presence of appropriate emotional events helps employees to overcome the job stressors (Gilbert et al., 2017). Although the impact of some mediating and moderating variables has been documented in the literature (Maslyn et al., 2017; Grewatsch and Kleindienst, 2018; Khan N. A. et al., 2019), the moderating impact of PS and WE in the POP and these job attitudes has not been investigated extensively. Our findings showed that the negative effect on job attitudes caused by POP can be mitigated by moderating constructs, i.e., PS and WE. These two personal resources enhance the workforce self-confidence that they can realize their job responsibilities, irrespective of hurtful political games (Shahani et al., 2019; Park and Lee, 2020); thus, employees are better capable of undertaking extra voluntary tasks and reflect positive behaviors that contribute to organizational productivity. Our study extends the prior research that suggests that desirable organizational outcomes such as efficiency could be accomplished by job resources that might alter the negative impact of POP on employee's outcomes.

Additionally, our study contributes by shedding some light on the debate whether POP promote or hinder organizational outcomes (Landells and Albrecht, 2015; Khan H. S. et al., 2019; Shahani et al., 2019). Our findings indicated that PS and WE are significant job resources that positively affect the job attitudes. Political skill proved to be an important resource that allows individuals to gain control and mastery over others (Bentley et al., 2017; Chiesa et al., 2019). In the same way, WE enables one to perform their duty whole-heartedly as employees having WE feel morally obligated to do their best. According to our results, these personal resources significantly reduce the adverse impact of POP on job attitudes.

### Practical Implications

An important practical implication of this study lies in the need for practitioners in organizations to essentially sharpen their ability to communicate with subordinates. Doing so will go a long way in enforcing the mutual relationships between superiors and subordinates. It may also serve as a platform for subordinates to diagnose and offer solutions to employee problems in the organization. Accordingly, this will ensure the job security of both subordinates and superiors and will result in higher productivity because there will exist a congenial atmosphere between superiors and subordinates.

This study indicated that PS can moderate the link between an extreme, agitation-based stressor and actions, as it was formerly associated with the dampening effects of stressors on attitudes and strain (Landells and Albrecht, 2019). Our findings suggest that in management or organizations facing challenging situations such as POP, the entities must conduct trainings on building skills such as PS in their workforce or interventions that enable the workforce to better manage their interpersonal resources, for instance, understanding different personality and motives of their colleagues (e.g., synergy or team building training sessions), learning how to manage their good name with workfellows and clients, or perhaps conducting sessions that help everyone to personally interact with each other personally in ways to build or restore trust.

To sum up, the WE of the workforce serve as a device for organizations to eradicate self-serving attitudes. Organizations could benefit from hiring an employee who is high in WE. This individual feature ought to be encouraged and needs to be developed for those currently working in the organization as well. Likewise, in order to improve workforce performance, organizations can educate their employees to manage difficult work conditions or identify various ways, for example, WE to lessen the odds of distressing conditions and their expected loss. With the purpose of encouraging such training programs, entities must underline a volunteering attitude that goes beyond the formal job responsibilities that could be advantageous for professional development and viewpoints of its employees. Lastly, our findings reveal that WE is an essential resource that benefits not only the individuals but also organizations. Productive trainings could invigorate the WE, and it is a noteworthy tool when workforce finds unfavorable situations at the workplace.

### Limitations and Future Studies

This study has some limitations. One important limitation lies in the study's domain. This is because the findings of the study cannot be generalized to all higher-education institutions worldwide. Thus, the findings of the study are limited to some selected higher-education institutions in Pakistan; hence, future studies should expand the scope to the regional and/or global level. Further, this study is cross sectional in nature; thus, future studies could be focused on time lag or be longitudinal. Moreover, the present study focused only on two personal resources; therefore, some other personal resources such as self-efficacy or psychological capital could be investigated in the current framework. Another limitation of the present study is that it was unable to examine the mediating effect of personal resources; thus, future research should consider this effect.

## Conclusion

The primary objective of the present study was to explore how POP affects job attitudes and how PS and WE moderate the relationship between POP and job attitudes in the education sector of Pakistan. Based on this, this study contributes to the existing literature in the domain of POP through the conceptual framework that was designed. The framework encapsulates the moderating impact of personal resources on the POP–job attitude relationship, which was missing from the initial framework in the POP literature pioneered by previous scholars (Ferris et al., 1989; Vigoda, 2002; Meisler and Vigoda-Gadot, 2014). In consideration of this, the findings of this study indicated that political practices negatively impact job attitudes in the higher-education sector in Pakistan, which is similar to the findings of Khan et al. (2017), Ahmed et al. (2020), and Asad et al. (2020). Furthermore, on the basis of the empirical findings, it is suggested that PS and WE have a significant positive impact on employees' attitudes toward the jobs assigned to them and aid in diffusing the negative effects of POP in the higher-education sector of Pakistan. Therefore, we suggest that managers can draw useful lessons from this study by putting in place mechanisms that bolster employees' skills and competence, consistent with the proposition by De Clercq and Belausteguigoitia (2020). Finally, it is imperative to appreciate that this study highlights the significance of coping with stress with the aid of tools such as PS and WE.

## Data Availability Statement

The raw data supporting the conclusions of this article will be made available by the authors, without undue reservation.

## Ethics Statement

The studies involving human participants were reviewed and approved by the study also complied with the guidelines by Higher Education Institutes and the statutory provisions in Pakistan for the conduct of research. Therefore, to conduct this study formal approval was sought from the Department of Air University School of Management (AUSOM), Air University, Islamabad Pakistan. The key objective of the study approval by the mentioned department was to highlight and confirm the diverse aspects of this investigation involving the feasibility, significance, ethical, and other associated perspectives. Furthermore, for the human participation in this research made it mandatory to seek ethical clearance which was granted by the same department. The patients/participants provided their written informed consent to participate in this study.

## Author Contributions

HK and SS conceptualized the study objectives and framework. HK designed the methodology and conducted the data analysis, while MZ, HW, and LM reviewed the manuscript. All authors contributed to the article and approved the submitted version.

## Conflict of Interest

The authors declare that the research was conducted in the absence of any commercial or financial relationships that could be construed as a potential conflict of interest.
